# Btk29A-Mediated Tyrosine Phosphorylation of Armadillo/β-Catenin Promotes Ring Canal Growth in *Drosophila* Oogenesis

**DOI:** 10.1371/journal.pone.0121484

**Published:** 2015-03-24

**Authors:** Noriko Hamada-Kawaguchi, Yasuyoshi Nishida, Daisuke Yamamoto

**Affiliations:** 1 Department of Developmental Biology and Neurosciences, Tohoku University, Graduate School of Life Sciences, Sendai, Japan; 2 Department of Biological Science, Graduate School of Science, Nagoya University, Nagoya, Japan; Technische Universität Dresden, GERMANY

## Abstract

*Drosophila* Btk29A is the ortholog of mammalian Btk, a Tec family nonreceptor tyrosine kinase whose deficit causes X-linked agammaglobulinemia in humans. The *Btk29A^ficP^* mutation induces multiple abnormalities in oogenesis, including the growth arrest of ring canals, large intercellular bridges that allow the flow of cytoplasm carrying maternal products essential for embryonic development from the nurse cells to the oocyte during oogenesis. In this study, inactivation of Parcas, a negative regulator of Btk29A, was found to promote Btk29A accumulation on ring canals with a concomitant increase in the ring canal diameter, counteracting the *Btk29A^ficP^* mutation. This mutation markedly reduced the accumulation of phosphotyrosine on ring canals and in the regions of cell-cell contact, where adhesion-supporting proteins such as *D*E-cadherin and β-catenin ortholog Armadillo (Arm) are located. Our previous *in vitro* and *in vivo* analyses revealed that Btk29A directly phosphorylates Arm, leading to its release from *D*E-cadherin. In the present experiments, immunohistological analysis revealed that phosphorylation at tyrosine 150 (Y150) and Y667 of Arm was diminished in *Btk29A^ficP^* mutant ring canals. Overexpression of an Arm mutant with unphosphorylatable Y150 inhibited ring canal growth. Thus Btk29A-induced Y150 phosphorylation is necessary for the normal growth of ring canals. We suggest that the dissociation of tyrosine-phosphorylated Arm from *D*E-cadherin allows dynamic actin to reorganize, leading to ring canal expansion and cell shape changes during the course of oogenesis.

## Introduction

Bruton’s tyrosine kinase (Btk) is a member of the Tec non-receptor tyrosine kinase family, which also includes Itk, Bmx, Tec, and Txk [1, 2]. Mutations in the *Btk* gene manifest as a severe immunodeficiency syndrome known as X-linked agammaglobulinemia (XLA) in humans and X-linked immunodeficiency (Xid) in mice [3]. Mammalian Btk is predominantly expressed in the B-cell lineage, at low levels in mature B lymphocytes and at higher levels in marrow-derived hematopoietic stem cells, common lymphoid progenitor cells and developing B cells. Indeed, Btk is involved in B-cell maturation [4, 5] and osteoclast differentiation [6, 7], as inferred from its expression profile. Differentiation of the B-cell lineage from hematopoietic stem cells to cells of the most mature stage, the plasma cells, consists of several discrete steps. Among these, the transition of pro-B cells into pre-B cells and the subsequent transition of pre-B cells into B lymphocytes are primarily blocked in XLA. However, the exact mechanism by which Btk mediates B-cell differentiation remains largely unknown [4, 8].

In *Drosophila*, the Tec kinase family is represented by the products of a single gene, *Btk29A* [9], conveniently simplifying the analysis of genotype-phenotype associations. The *Btk29A* gene produces two types of transcripts, type 1 and type 2. The type 2 product is considered to be the ortholog of mammalian Btk, since it possesses all functional domains common to mammalian Btk, i.e., the PH, TH, SH3, SH2 and kinase domains [9]. The type 1 product, in contrast, lacks the entire PH domain and part of the TH domain, and instead has a short stretch of a unique sequence [9]. The mutations in the *Btk29A* locus lead to developmental defects in a wide variety of tissues, such as failures in blastoderm cellularization [10], invagination of salivary gland placodes [11], dorsal closure [12], male genital formation [9, 13] and oogenesis [14–17]. Although the tissues affected by *Btk29A* mutations are spectacularly divergent, most, if not all, of these phenotypes appear to result from a deficit in actin organization [10, 11, 14].

To elucidate how Btk29A regulates the morphogenesis of actin-based structures, we focused our attention on the growth of ring canals, the actin-rich intercellular pores connecting 16 sibling germ cells, i.e., 15 nurse cells and an oocyte [18]. Maternal products, including the morphogens governing the formation of embryonic body axes, are transferred from nurse cells to the oocyte across the ring canals; the malfunction of these canals could thus lead to serious impairments in early embryogenesis [18, 19].

**Fig 1:**
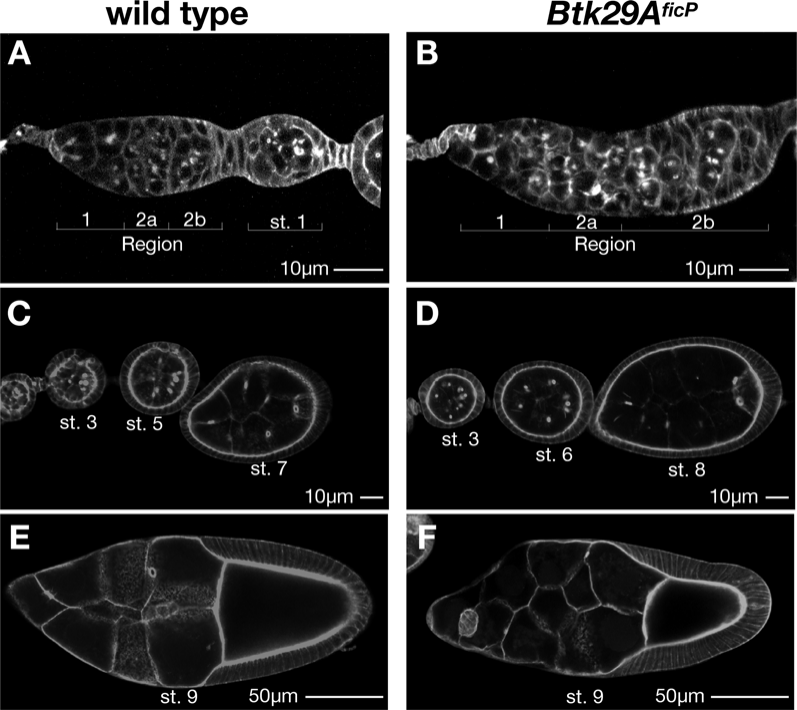
Ovarian phenotypes of *Btk29A* ^*ficP*^ mutants. (**A** and **B**) Germaria and early egg chambers of the wild type (**A**) and *Btk29A* ^*ficP*^ (**B**) stained for phalloidin. In the wild type, Region 2b is bordered posteriorly by elongated follicle cells; in *Btk29A* ^*ficP*^ mutants, these cells are interspersed with germ cells having a round appearance, reflecting a wrapping defect. The number of germ cells present in a germarium is variable and the overall shape of the germarium is distorted in *Btk29A* ^*ficP*^ mutants, compared with that of the wild type. At stage 1, a wild-type egg chamber is always oval in shape and invariably contains 16 germ cells. (**C** and **D**) Stage 3—stage 8 egg chambers of wild-type (**C**) and *Btk29A* ^*ficP*^ mutant (**D**) ovaries. (**E** and **F**) Stage 9 mature egg chambers of wild-type (**E**) and *Btk29A* ^*ficP*^ (**F**) ovaries. Scale bars: 10 μm for (**A**-**D**) and 50 μm for (**E** and **F**).

All germ cells are produced by germ stem cells (GSCs) in the germarium, which is subdivided into Regions 1–3 (Region 3 corresponds to the stage 1 egg chamber; see below and Fig. 1A). A GSC divides asymmetrically to generate a GSC and a cystoblast (CB). A CB undergoes 4 rounds of symmetrical divisions, resulting in incomplete cytokinesis and producing a cyst composed of 16 connected cells. 15 cells in the cyst become nurse cells and the remaining posterior cell takes on the oocyte fate. Ring canals are derivatives of the arrested contractile rings resulting from incomplete cytokinesis, in which a contractile ring does not close so that two sib-cells remain connected with a canal. Thus the first ring canal emerges at the first division of the CB in Region 1 of the germarium (Fig. 1A), and two subsequent divisions also take place and produce corresponding ring canals. Other additional ring canals are the products of further incomplete cytokineses which occur in Region 2a and Region 2b (Fig. 1A). In Region 2b, the cyst changes shape and becomes a one cell-thick disc that spans the whole width of the germanium. A cyst in Region 2b continues to develop into a stage 1 egg chamber encapsulated by the follicular layer (Fig. 1A). The egg chamber matures through stages 1–12 (Figs. 1A, 1C and 1E for stages 1–9). Ring canals are therefore composed of molecules constituting contractile rings, the primary component of which is actin.

Previous studies demonstrated that phosphotyrosine is accumulated on the ring canals in a *Btk29A*- and *src64-*dependent manner, and that the loss of *Btk29A* leads to a marked reduction in the ring canal size [15, 17]. Although phosphorylated Kelch, an actin-filament cross-linking protein [20], partly contributes to accumulated phosphotyrosine on the ring canal, the absence of Kelch has little effect on the early phase of ring canal growth [21]. In contrast, the loss of *Btk29A* induces growth arrest at the early stage of ring canal development [15]. In an effort to decipher the molecular mechanism whereby Btk29A regulates oogenesis, we have identified Armadillo (Arm), the *Drosophila* ortholog of β-catenin, as a unique *in vitro* and *in vivo* substrate for Btk29A [22]. Here we show that two conserved tyrosine residues of Arm, Arm Y150 and Arm Y667, are strongly phosphorylated by Btk29A in ring canals. We postulate that Btk29A-induced tyrosine phosphorylation facilitates the dissociation of Arm from adherens junctions, thereby altering the actin organization of ring canals, presumably by modulating the activity of actin-binding proteins. We further show that Parcas (Pcs), the fly ortholog of Sab that was identified as a negative regulator of Btk in mammals, counteracts the Btk29A action in ring canals, representing a conserved regulatory mechanism for Btk29A.

## Materials and Methods

### Flies

Flies were raised on cornmeal-agar-yeast media at 25°C. Canton-Special (CS) was used as a control strain. The *Btk29A* ^*ficP*^ allele was isolated in one of our laboratories [9]. Other fly lines were obtained from the Bloomington Stock Center, Drosophila Genetic Resource Center (Kyoto, Japan) and Viena Drosophila RNAi Center.

### Histology

For antibody staining, ovaries were dissected in PBS and immersed in 4% paraformaldehyde in PBS for 30 min. The ovaries were washed three times in PBT, blocked for 1.5 hr in PBS supplemented with 1% Triton and 0.1% BSA, and then incubated with a primary antibody for 3 hrs at room temperature or at 4°C overnight. The primary antibodies used in this study were anti-Btk29A (1:10) [14], anti-Arm (1:10; Developmental Studies Hybridoma Bank), anti-phosphotyrosine 4G10 (1:250; Upstate Biotechnology), anti-pY142 (1:200; ECM Biosciences) and anti-pY654 (1:200; Invitrogen). The fluorescence-conjugated secondary antibodies were purchased from Molecular Probes and used at a 1:250 dilution. Texas Red-X phalloidin was purchased from Molecular Probes and used at a 1:50 dilution. All samples were mounted in 80% glycerol. Images were obtained with a Zeiss LSM 510 META confocal microscope using Zeiss LSM Image Browser and processed with Adobe Photoshop software. We measured the longest distance across the lumina of a ring canal to define its diameter using Adbe Photoshop software. Statistical treatments of data were carried out with Microsoft Exel Analysis Toolpak.

## Results

### Btk29A is Required for Ring Canal Growth

**Fig 2:**
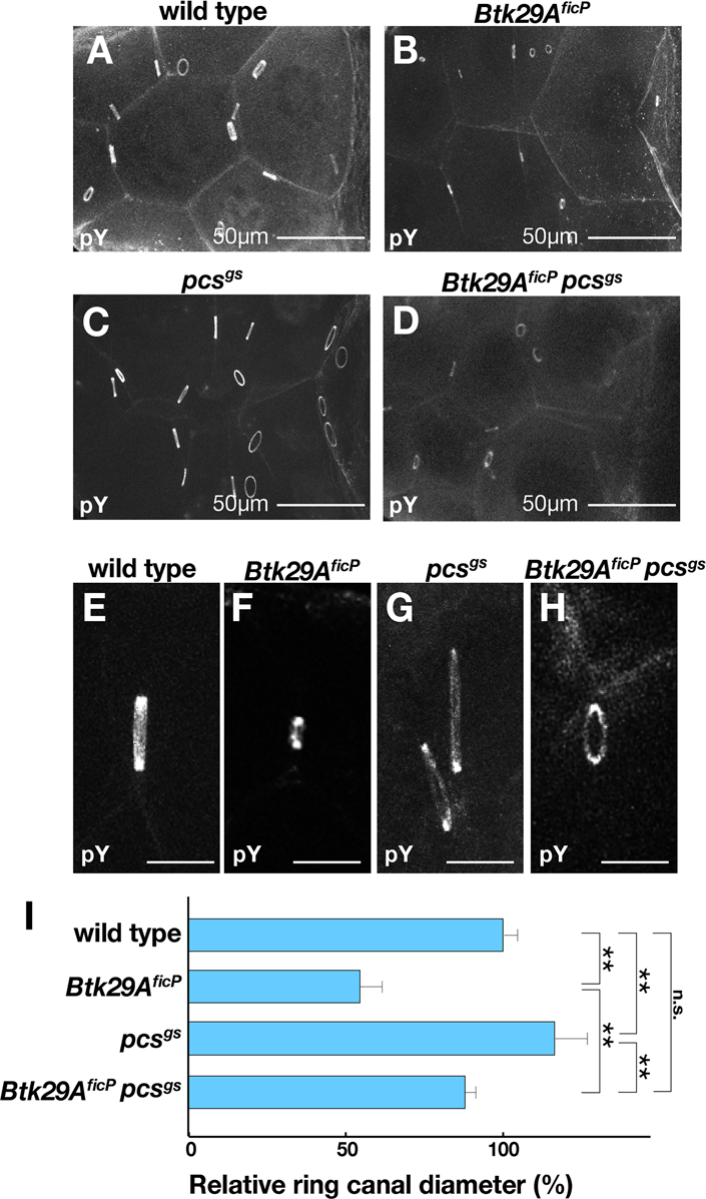
Ring canal growth is arrested in *Btk29A* ^*ficP*^ mutants. **(A—D)** Anti-phosphotyrosine 4G10 antibody staining highlights the ring canals of mature egg chambers from the wild type (**A**), *Btk29A* ^*ficP*^ (**B**), *pcs* ^*gs*^ (**C**) and *Btk29A* ^*ficP*^ *pcs* ^*gs*^ (**D**). Scale bars: 50 μm. (**E—H**) Lateral views of representative ring canals in the stage 9 egg chambers of the wild type (**E**), *Btk29A* ^*ficP*^ (**F**), *pcs* ^*gs*^ (**G**) and *Btk29A* ^*ficP*^ *pcs* ^*gs*^ (**H**) after staining with the anti-phosphotyrosine antibody. Scale bars: 10 μm. (**I**) Quantitative comparisons of the ring canal diameter among different genotypes: wild type (n = 143), *Btk29A* ^*ficP*^ (n = 339), *pcs* ^*gs*^ (n = 282), and *Btk29A* ^*ficP*^ *pcs* ^*gs*^ (n = 40). The diameters of individual ring canals at stage 9 were normalized by the A/P (long) axis of each egg chamber, and the values relative to those of the wild-type control (the means ± standard errors in %) are shown for the respective genotypes. Statistical differences between the indicated pairs were evaluated by Student’s *t*-test (**p<0.01).

**Fig 3:**
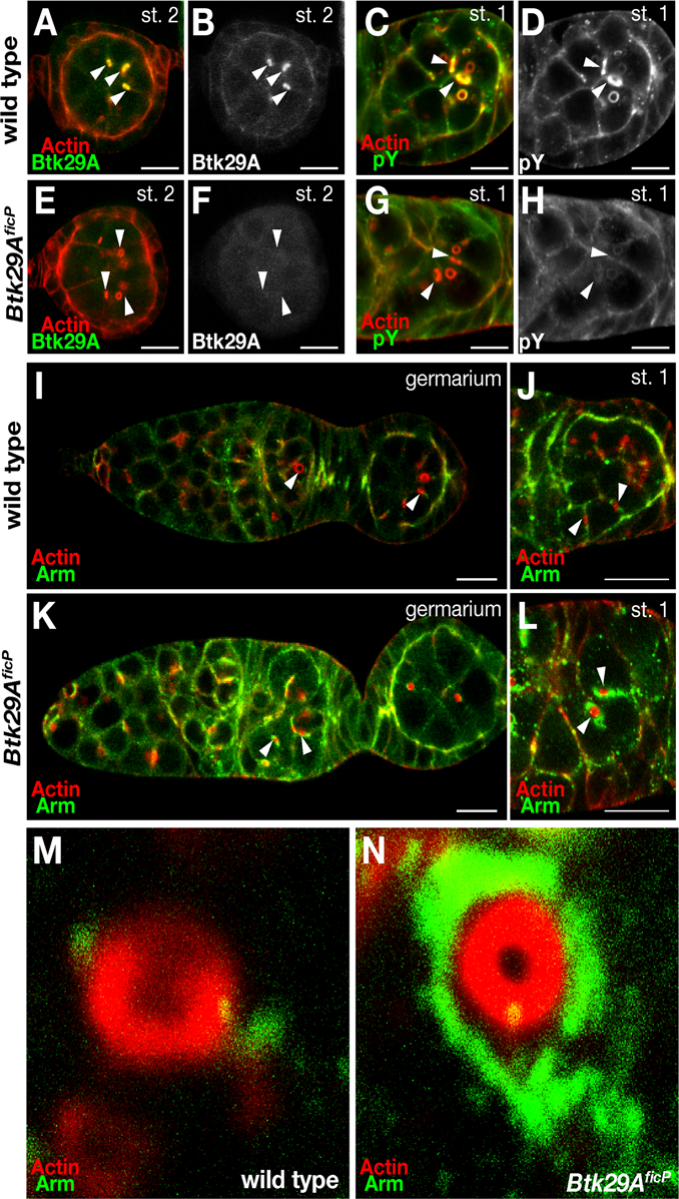
Effect of the *Btk29A* ^*ficP*^ mutation on the localization of Btk29A, Arm and phosphotyrosine. The panels show the stage 2 (**A, B, E** and **F**) and the stage 1 (**C, D, G** and **H**) egg chambers from wild-type (**A—D**) and *Btk29A* ^*ficP*^ mutant (**E—H**) ovaries doubly stained with phalloidin for actin (red in **A, C, E** and **G**) and the anti-Btk29A antibody (green in **A** and **E**) or the anti-phosphotyrosine antibody (green in **C** and **G**). The images of antibody staining are also shown in black and white in (**B**), (**D**), (**F**) and (**H**) to aid in comparisons of the localization and abundance of Btk29A or phosphotyrosine between the wild-type and *Btk29A* ^*ficP*^ mutant egg chambers. Btk29A is highly enriched around ring canals and cell-cell contact regions in the wild-type chambers, whereas it is barely detectable in the *Btk29A* ^*ficP*^ mutant chambers. (**I—N**) Arm (green) is localized along the cell-cell contact regions and ring canals in the wild-type (**I**) and *Btk29A* ^*ficP*^ mutant (**K**) germaria. Actin is visualized by phalloidin staining (red). The entire stage 1 (**J** and **L**) and close-up views of ring canals in stage 1 (**M** and **N**) of the wild-type (**J** and **M**) and *Btk29A* ^*ficP*^ mutant (**L** and **N**) germaria are shown. Some of the ring canals are marked with arrowheads in (**A**—**L**). Note the marked accumulations of Arm in the regions surrounding the ring canals in *Btk29A* ^*ficP*^ mutants. Scale bars: 10 μm.

It has been reported that germline clones for strong mutant alleles in the *Btk29A* locus display several distinct phenotypes, such as ring canal undergrowth [15, 17], fusome distortion [14], defects in karyosome formation [14], packaging defects [14], aberrant border cell migration (N. Hamada-Kawaguchi, unpublished data) and oocyte mislocalization [15]. In keeping with these observations, *Btk29A* ^*ficP*^ mutant ovaries, expressing only the short type 1 splice form of Btk29A, were grossly aberrant as a result of the irregular shapes and sizes of germ cells and follicle cells (Figs. 1B, 1D and 1F). Moreover, the *Btk29A* ^*ficP*^ egg chambers were equipped with ring canals that were much smaller than those of the wild type (Figs. 2A, 2B, 2E and 2F): mature wild-type egg chambers have ring canals of 8–10 μm in diameter (Fig. 2I), whereas those of *Btk29A* ^*ficP*^ were about 3–5 μm in diameter (Fig. 2I), as reported for other *Btk29A* alleles that affect both type 1 and type 2 products ([15, 17] and N. Hamada-Kawaguchi, unpublished data). It is notable that the lumina of mutant ring canals appeared to be very narrow in *Btk29A* ^*ficP*^ mutants, compared to the wild type (Figs. 3M and 3N).

The *pcs* gene encodes a *Drosophila* homolog of Sab (an SH3 domain-binding protein associated preferentially with Btk; [23, 24], which is a negative regulator of Btk in mammals [25]. The average diameter of ring canals differed significantly among the wild type, *Btk29A* ^*ficP*^ homozygotes, and *pcs* ^*gs*^ homozygotes (Fig. 2I). The egg chambers null for *pcs* (*pcs* ^*gs*^; [24]) had extremely large ring canals (Figs. 2C, 2G and 2I), while *Btk29A* ^*ficP*^ *pcs* ^*gs*^ double-mutant females developed ring canals that were only slightly smaller (p<0.05 in the Student’s *t*-test) than the wild-type ones (Figs. 2D, 2H and 2I). These observations reinforce the hypothesis that Btk29A plays a pivotal role in ring canal development.

### Btk29A Regulates Arm Subcellular Localization

To evaluate the importance of kinase activity in the developmental role of Btk29A, wild-type and *Btk29A* ^*ficP*^-mutant egg chambers were subjected to immunostaining with anti-Btk29A and anti-phosphotyrosine antibodies (Figs. 3C, 3D, 3G and 3H). We found that anti-Btk29A-reactive materials (Figs. 3A, 3B, 3E and 3F) and anti-phosphotyrosine antibody-reactive materials (Figs. 3C, 3D, 3G and 3H) were both enriched in ring canals as well as cell borders in wild-type egg chambers. Indeed, anti-phosphotyrosine staining in all these regions decreased dramatically in *Btk29A* ^*ficP*^-mutant egg chambers (Figs. 3G and 3H). Unlike in the previous report on *Btk29A* ^*k05610*^ [15], residual anti-phosphotyrosine staining was observed, possibly due to type 1 activities remaining in *Btk29A* ^*ficP*^. The regions accumulating phosphotyrosine in egg chambers seem to match the known distribution of adherens junctions [26], through which the *D*E-cadherin-β-catenin complex communicates with the cytoskeletal actin network. However, immunohistochemical examination revealed that the localization and abundance of *D*E-cadherin were marginally affected by the *Btk29A* ^*ficP*^ mutation (data not shown). In contrast, staining for Arm, an important signaling component of the *D*E-cadherin complex, was remarkably more intense around the ring canals and cellular junctions of *Btk29A* ^*ficP*^-mutant egg chambers (Figs. 3I - 3N). This observation strongly suggests that Btk29A might control morphogenetic events by phosphorylating a specific component of the *D*E-cadherin complex. An obvious candidate for the Btk29A substrate is Arm, which has been shown to be a direct phosphorylation target of Btk29A in ovaries [22].

### Btk29A Phosphorylates Arm *In Vivo*

To determine whether Arm associated with ring canals is phosphorylated by Btk29A in developing oocytes, we employed the anti-pY142 and anti-pY654 antibodies raised against mammalian β-catenin for the detection of phosphorylated *Drosophila* Arm, based on the fact that the sequences around the potential phosphorylation sites are identical between β-catenin and Arm and thus are recognized by the same antibodies [22].

**Fig 4:**
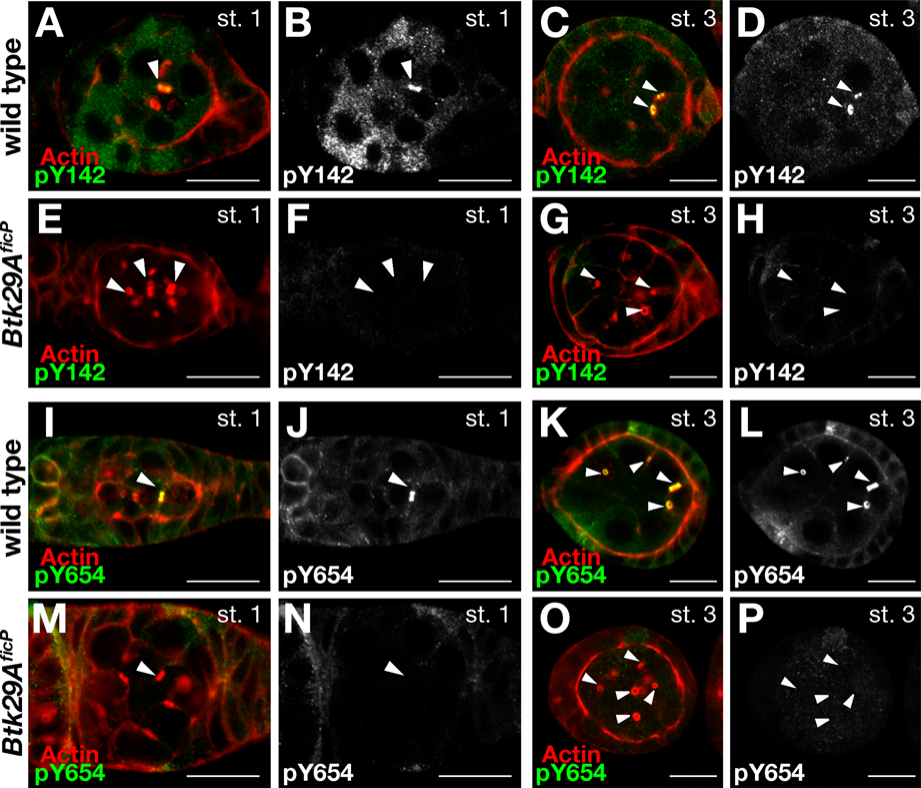
Btk29A-dependent tyrosine phosphorylation of Arm associated with ring canals. Staining with the anti-pY142 (**A—H**) or anti-pY654 (**I**—**P**) antibody highlights the ring canals of wild-type egg chambers at stage 1 (**A, B, E, F** and **I, J, M, N**) and at stage 3 (**C, D, G, H** and **K, L, O, P**). In the Region 2b germarium and stage 1 egg chamber, a single ring canal is stained by the anti-pY142 or anti-pY654 antibody (**A**, **B**, **I** and **J**). At later stages, additional ring canals of wild-type egg chambers become positive for the anti-pY142 (**C** and **D**) and anti-pY654 (**K** and **L**) antibodies (stage 3 egg chambers are shown). Note that β-catenin Y142 and Y654 are equivalent to Arm Y150 and Y667, respectively. In *Btk29A* ^*ficP*^ mutants, immunoreactivity to the anti-pY142 (**E**, **F**, **G** and **H**) and anti-pY654 (**M**, **N**, **O** and **P**) antibodies is almost completely absent. Some of the ring canals are marked with arrowheads in (**A**)–(**P**). Scale bars: 10 μm. The stage 1 and 3 egg chambers were doubly stained with phalloidin for actin (red) and the anti-pY142 antibody (green, **A**, **C**, **E** and **G**) or the anti-pY654 antibody (green, **I**, **K**, **M** and **O**). Images of staining with the anti-pY142 antibody (**B**, **D**, **F** and **H**) or the anti-pY654 antibody (**J**, **L**, **N** and **P**) are also shown in black and white to aid in comparisons between the wild-type and *Btk29A* ^*ficP*^ egg chambers. Scale bars: 10 μm.

**Fig 5:**
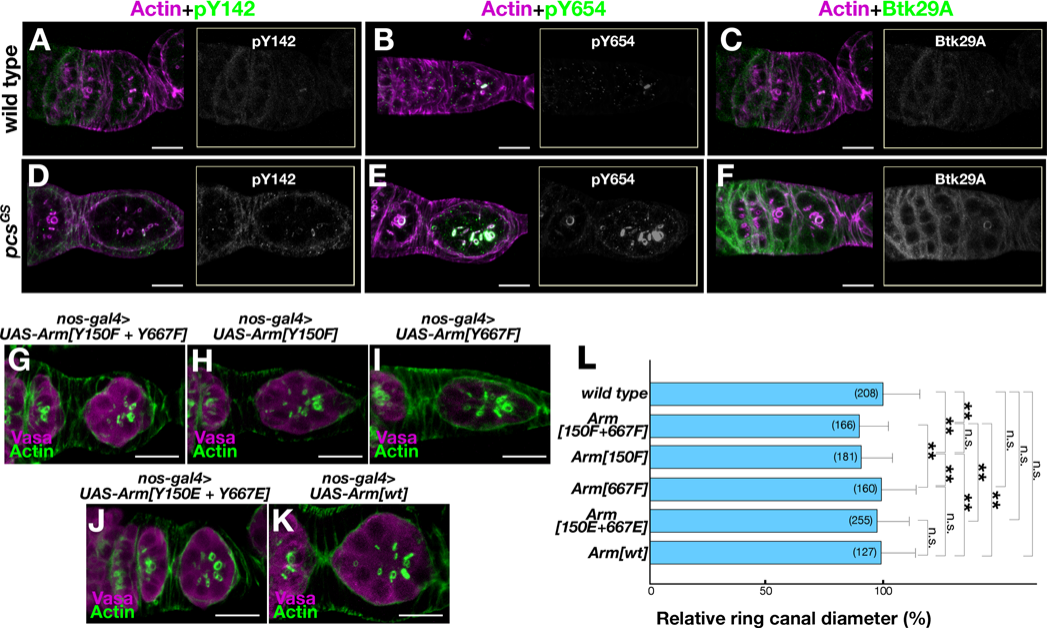
Effects of the *pcs* mutation and *arm*-variant overexpression on ring canal development. (**A**—**F**) Stage 1-egg chambers were stained with antibodies against pY142 (**A** and **D**), pY654 (**B** and **E**) or Btk29A (**C** and **F**) in the wild-type (WT; **A**—**C**) and *pcs* ^*gs*^ mutant (**D**—**F**) ovaries. (**G**—**K**) Effects of overexpression of Y150F+Y667F (**G**), Y150F (**H**), Y667F (**I**), Y150E+Y667E (**J**), or *arm* ^*+*^ (**K**) on ring canal growth. Scale bars: 10 μm. (**L**) Quantitative comparisons of the ring canal diameter at stage 1 among different genotypes: wild type (n = 208), Y150F+Y667F (n = 166), Y150F (n = 181), Y667F (n = 160), Y150E+Y667E (n = 255), and *arm* ^*+*^ (n = 127). The means ± standard errors of ring canal diameters (in μm) at stage 1 are shown for the respective genotypes. The numbers of ring canals examined are shown in parentheses. Statistical differences between the indicated pairs were evaluated by Student’s *t*-test (**p<0.01; n.s., nonsignificant).

In wild-type ovaries, strikingly intense immunoreactivity to the anti-pY142 antibody was observed in a single ring canal in Region 2b (not shown) and the stage-1 egg chamber (Fig. 4A and 4B). Subsequently, other ring canals became progressively positive to the anti-pY142 antibody (Figs. 4C and 4D), until ultimately all ring canals were intensely stained by the antibody. The ring canal that first became immunopositive to the anti-pY142 antibody had a larger diameter than other ring canals, indicating that it is associated with the oocyte membrane [27]. The anti-pY654 antibody labeled ring canals in a pattern similar to that of the anti-pY142 antibody (Figs. 4I - 4L). Moreover, the immunoreactivity to the anti-pY142 and anti-Yp654 antibodies of cellular boundaries was less intense than that of ring canals (Figs. 4A-4D and 4I-4L). In contrast, the intensity of staining by the anti-phosphotyrosine antibody 4G10, which reflects the overall phosphorylation status to which other tyrosine kinases also contribute [28], was similar in cellular boundaries and ring canals (Figs. 3A-3H), revealing the specificity in the localization of phosphorylated Arm. Importantly, the intensity of staining for phosphorylated Arm was dramatically reduced in *Btk29A* ^*ficP*^ mutant egg chambers (Figs. 4E - 4H and 4M - 4P). Notably, the immunoreactivity of ring canals to anti-pY142 and anti-pY654 antibodies in *pcs* mutants appeared earlier than in the wild type; all ring canals in *pcs* mutants were intensely stained with these antibodies in stage 1 egg chambers, where only the largest ring canal was immunopositive in the wild type (Figs. 5A, B, D and E). Furthermore, the staining intensity of ring canals by an anti-Btk29A antibody was remarkably stronger in *pcs* than wild-type ovaries (Figs. 5C and 5F). These observations strengthen the hypothesis that two tyrosine residues of Arm, Y150 and Y667, are phosphorylated by Btk29A at the early stage of ring canal growth, and Pcs negatively regulates this Btk29A function. Although it has been reported that the mammalian Pcs ortholog Sab inhibits the catalytic activity of Btk [25], our result implies that Pcs might act as a negative regulator of Btk29A by a distinct mechanism—for example, by promoting Btk29A degradation.

### Impaired Arm (β-catenin) Phosphorylation Impedes Ring Canal Growth

To explore roles of Arm phosphorylation in ring canal growth, we overexpressed the unphosphorylatable and phosphomimetic forms of Arm in germ cells as driven by *nos-GAL4*. Since ring canals grow rapidly while dynamically changing their orientation, accurate staging of germ cell development and complete reconstitution of the three-dimensional structure are critical for a precise estimate of their diameters. The effects of overexpression of Arm variants could be more subtle than those induced by mutations in the genome. We therefore decided to measure the ring canal diameter at stage 1, as this stage is unequivocally determined by the completion of follicular encapsulation, which is immediately preceded by the formation of a one cell-thick disc of germ cells that spans the whole width of the germanium. Overexpression of Y150F+Y667F, in which both of the tyrosine residues of Arm were replaced with unphosphorylatable phenylalanine, led to a significant reduction of the diameter of ring canals compared with that of the wild type (Figs. 5G and 5L). Overexpression of Y150F, in which only Y150 was made unphosphorylatable while Y667 remained intact, was similarly effective in reducing the ring canal diameter, whereas Y667F overexpression was without significant effect (Figs. 5H, 5I and 5L), indicating that tyrosine phosphorylation at Y150, rather than Y667, is critical for normal ring canal growth. Interestingly, overexpression of the phosphonomimetic variant Y150E+Y667E neither increased nor decreased the ring canal diameter significantly (Figs. 5J and 5L). These results seem to suggest that Y150 phosphorylation is necessary but not sufficient for normal ring canal growth, which tolerates wide variations in the amount of Arm in a cell.

## Discussion

We present evidence that phosphorylation of Arm Y150 and Arm Y667 by Btk29A is a critical step in the growth of ring canals, which mediate the transport of maternal materials required for embryogenesis after the fertilization of oocytes. First, the *Btk29A* ^*ficP*^ mutation arrests ring canal growth before stage 5 (Fig. 1 and [29]). Second, Btk29A and Btk phosphorylate, *in vivo*, tyrosine residues of Arm and β-catenin, respectively [22]. Third, the antibody that specifically recognizes Y142- or Y654-phosphorylated β-catenin strongly labels Arm pY150 or pY667 associated with ring canals in a Btk29A-dependent manner (Fig. 4). Fourth, the level of phosphorylation at Arm Y150 and Arm Y667 in ovarian lysates dramatically decreases in *Btk29A* ^*ficP*^ mutants [22]. Fifth, overexpression of Arm mutants devoid of phosphorylation at Y150 inhibited the growth of ring canals (Fig. 5H). We have also demonstrated that Pcs is an important negative regulator of Btk29A: loss of Pcs stimulated accumulation of Btk29A on ring canals, resulting in the induction of premature tyrosine phosphorylation of Arm and the enlargement of ring canals (Figs. 5A-5F). Although loss of the Btk29A inhibitor Pcs was sufficient for inducing ring canal overgrowth, overexpression of a putative phosphomimetic form of Arm was unable to produce a similar effect. This might suggest the involvement of unknown substrates of Btk29A other than Arm in the regulation of ring canal growth.

In mammalian cultured cells, the impact of tyrosine phosphorylation of β-catenin on its transcriptional activity and cell adhesion has been documented [30]. For example, an increase in the tyrosine phosphorylation of β-catenin by v-Src induces a rapid loosening of cell-cell contact and promotes invasiveness [31]. Treatment of cells with a tyrosine-phosphatase inhibitor leads to the redistribution of β-catenin from cellular junctions to cytoplasm or the nucleus [31].

A computer-aided search for the consensus phosphorylation sites for Btk and related kinases revealed two primary candidate residues in Arm: Y150 and Y667, which correspond to Y142 and Y654 in mammalian β-catenin, respectively [32]. An additional residue that could be phosphorylated in human β-catenin is Y86, which is not conserved in Arm. We previously showed that Btk29A and Btk phosphorylate at least two tyrosine residues, Y150 and Y667 in Arm, and Y142 and Y654 in mammalian β-catenin, respectively [22]. Y142 phosphorylation was shown to be important in allowing β-catenin to bind to α-catenin [30], which regulates the mobility of the *D*E-cadherin-β-catenin complex [33]. The phosphorylation of Y142 in β-catenin was reported to be critical for binding to the transcriptional cofactor, BCL9-2 [34], a human paralog of which is mutated in B-cell lymphoma, although this finding has not been successfully reproduced [35]. On the other hand, Y654 is placed in a domain for binding to the basal transcription factor TATA-binding protein (TBP) [36].

Ring canals are derivatives of arrested meitotic cleavage furrows and thus contain abundant F-actin. They also contain mucin-like glycoprotein [37], the Adductin homolog Hu-li tai shao (Hts) [38], cortactin [39], ABP280/filamin [40, 41], Kelch [21] and Src64 and Btk29A tyrosine kinases [17, 29]. Cortactin, ABP280/filamin and Kelch are F-actin-binding proteins, while Hts regulates the subcellular localization of F-actin. In fact, F-actin is the core component of ring canals. For ring canal growth, actin filaments seem to be polymerized at the plasma membrane to expand the ring canal rim and are disassembled at the cytoplasmic face to maintain the lumen [20]. The Arp2/3 complex is suggested to function in the polymerization of actin filaments at the ring canal plasma membrane to drive ring canal growth [42]. Interestingly, Godt and Tepass [26] have shown that the *D*E-cadherin complex is accumulated at cell membranes surrounding the ring canals in germarium Region 2b and the stage 1 follicle. It is also known that ring canals fail to develop properly in germline clones for *arm* mutations [43]. The E-cadherin-β-catenin complex is associated with the F-actin network, and this association is known to be mediated by certain actin-binding proteins. Namely, α-catenin mediates the association of the E-cadherin-β-catenin complex with dynamic F-actin to regulate the mobility of adhesive junctional foci, while some other mediators are involved in the association of the E-cadherin-β-catenin complex with stable F-actin [33].

For the rapid growth of ring canals, dynamic F-actin must be polymerized and depolymerized while tethered to the plasma membrane. Btk29A-mediated tyrosine phosphorylation of Arm seems to represent a novel mechanism regulating this process: dissociation of Arm from *D*E-cadherin upon its tyrosine phosphorylation by Btk29A might confer more flexibility on the association of *D*E-cadherin/Arm with F-actin, thus making possible the reorganization of F-actin in ring canals while the *D*E-cadherin complex is anchored to the appropriate sites on the germ-cell plasma membrane. On the other hand, Piedra et al. [44] have shown that activated Fer or Fyn phosphorylates β-catenin at Y142, resulting in the loss of its association with α-catenin. Tyrosine phosphorylation of β-catenin/Arm by Btk/Btk29A could similarly increase free α-catenin, which, in turn, might suppress Arp2/3-mediated actin polymerization by competing with Arp2/3 for binding to actin filaments [45].

The release of tyrosine-phosphorylated β-catenin/Arm from adherens junctions increases the cytoplasmic pool of β-catenin/Arm that is readily available, upon receiving a Wnt signal, for translocation to the nucleus, where it regulates transcription [22, 30]. It remains to be determined whether or not any changes in transcription due to Btk29A-mediated tyrosine phosphorylation of Arm contribute to ring canal growth.
